# Establishment, Growth, and Yield Potential of the Perennial Grass *Miscanthus × Giganteus* on Degraded Coal Mine Soils

**DOI:** 10.3389/fpls.2017.00726

**Published:** 2017-06-12

**Authors:** Stanisław Jeżowski, Michal Mos, Sam Buckby, Joanna Cerazy-Waliszewska, Wojciech Owczarzak, Andrzej Mocek, Zygmunt Kaczmarek, Jon P. McCalmont

**Affiliations:** ^1^Institute of Plant Genetics, Polish Academy of SciencesPoznań, Poland; ^2^Energene Sp. z o.o.Łódź, Poland; ^3^Terravesta Ltd, Cedar FarmLincoln, United Kingdom; ^4^Department of Soil Sciences and Land Protection, Poznan University of Life SciencesPoznań, Poland; ^5^Institute of Biological, Environmental and Rural Sciences, Aberystwyth UniversityAberystwyth, United Kingdom

**Keywords:** *Miscanthus*, biomass, phyto-remediation, soil reclamation, brown fields

## Abstract

*Miscanthus × giganteus* is a giant C4 grass native to Asia. Unlike most C4 species, it is relatively cold tolerant due to adaptations across a wide range of altitudes. These grasses are characterized by high productivity and low input requirements, making them excellent candidates for bioenergy feedstock production. The aim of this study was to investigate the potential for growing *Miscanthus* on extremely marginal soils, degraded by open lignite (brown coal) mining. Field experiments were established within three blocks situated on waste heaps originating from the lignite mine. Analyses were conducted over the first 3 years following *Miscanthus* cultivation, focusing on the effect of organic and mineral fertilization on crop growth, development and yield in this extreme environment. The following levels of fertilization were implemented between the blocks: the control plot with no fertilization (D0), a plot with sewage sludge (D1), a plot with an identical amount of sewage sludge plus one dose of mineral fertilizer (D2) and a plot with an identical amount of sewage sludge plus a double dose of mineral fertilizer (D3). Crop development and characteristics (plant height, tillering, and biomass yield [dry matter]) were measured throughout the study period and analyzed using Analysis of Variance (ANOVA). Significant differences were apparent between plant development and 3rd year biomass production over the course of the study (0.964 kg plant^-1^ for DO compared to 1.503 kg plant^-1^ for D1). Soil analyses conducted over the course of the experiment showed that organic carbon levels within the soil increased significantly following the cultivation of *Miscanthus*, and overall, pH decreased. With the exception of iron, macronutrient concentrations remained stable throughout. The promising yields and positive effects of *Miscanthus* on the degraded soil suggests that long term plantations on land otherwise unsuitable for agriculture may prove to be of great environmental and economic significance.

## Introduction

There is growing European and global interest in the share of green energy within the overall energy budget of member states. In accordance with the recommendations of the European Commission, the European Union agreed to an increased contribution to total energy from renewables to on average of 20% by 2020 (The European Parliament, and the council of the European Union, 2009). Of this 20%, 60% is to be sourced from perennial energy crops (e.g., *Miscanthus*, willow, poplar, etc.), without impacting on food production. This implies that such energy crops are to be grown on more marginal agricultural land (FAO, 1999; Hastings et al., 2009; Communication from the Commission to the European Parliament, 2014), typically of poor quality and unsuitable for conventional crop cultivation. Such land is often a result of contamination through industrial activity, e.g., heavy metal burdens or general degradation by mining. In Poland, primary areas of concern are waste heaps left after opencast lignite mining. The reclamation process for such areas can be extremely challenging and prolonged since soils in those heaps are mineral, sterile rocks lacking the organic layer required to provide an optimal environment for plant growth and development.

In response to these challenges, this study tests the growth potential of *M. × giganteus (M×g)* as an aid to reclamation of open cast lignite mining areas. *M×g* is a high yielding, low input, perennial, giant grass, belonging to the group of C4 carbon pathway plants (Jeżowski, 1994, 2001; Deuter and Jeżowski, 2000; Heaton et al., 2004; Sacks et al., 2013). The potential of the crop for biomass, bioenergy and biofuel production is widely recognized (El Bassam, 1997; Deuter and Jeżowski, 1998; Lewandowski, 2006; Hastings et al., 2008; Faber and Kuś, 2009; Chung and Kim, 2012; Clifton-Brown et al., 2013). Moreover, *Miscanthus* can play a useful role in improving soil structure and levels of organic matter (Jeżowski, 1994; Majtkowski, 1998; Faber et al., 2007). The crop is characterized by extensive root/rhizome networks, that can reduce soil compaction and allow a greater water buffering capacity (Wanat et al., 2013). In addition, the plants can input relatively high levels of organic material into the soil each year (Faber et al., 2007). Approximately 30% of the total annual biomass production (leaf litter drop) will fall to the ground over winter (Lewandowski et al., 2000; Clifton-Brown et al., 2001); a significant proportion of this is re-cycled into the soil as organic matter (Hansen et al., 2004; McCalmont et al., 2017).

The primary aim of this study was to investigate the potential for growth, development and yield of *M×g* in the first 3 years following cultivation in the poor soil conditions of post mining waste heaps. The following hypotheses were tested:

H_1_: *M×g* can be successfully established and produce viable yields on post mining soils after the third year of growth following preliminary reclamation measures prior to planting.
H_0_: Post mining land in the first few years of reclamation is not suitable for the successful establishment of *M×g*.

Additionally, it was intended to determine if the plantation of the grasses on such soils could play a useful, or even profitable role, in the reclamation of these soils following appropriate cultivation and fertilization procedures.

## Materials and Methods

### Plant Material

The planted material was the clonal, *Miscanthus × giganteus (M×g)*; a naturally occurring sterile hybrid formed through the crossing of *M. × sinensis* and *M. × sacchariflorus* (Greef and Deuter, 1993). The initial material for selection was a clone of *M×g* imported to Poznań in 1998 by the Institute of Plant Genetics, the Polish Academy of Sciences (IPG PAS) from TINPLANT GmbH in Klein Wanzleben (Germany). The best plants, selected by their yields of biomass and reproduced using rhizomes, are still growing today in the collection plot of IPG PAS.

### Field Trials

Growth and yield potential of the *M×g* plants was assessed during the period from May 2012 to February 2015 at field trials established in reclaimed areas of waste heaps at the Adamów open lignite mine near Turek. This site is located at the eastern side of the Wielkopolska region (53° 43′ N, 18° 41′ E), consisting largely of mine spoil arranged in biologically inactive heaps containing few plant available nutrients. There is a distinct contrast between these sites and natural soils, which have organic, biologically active upper layers; post-mining soils, at least in the first few years, contain no organic compounds. As such, preliminary reclamation measures were applied 3 years prior to establishment. In spring of 2009, a legume mix (*Medicago sativa* ssp. *sativa, Medicago sativa* ssp. *media (M × varia)* Martyn) was planted at the site in an attempt to establish a suitable soil for the cultivation of *M×g*.

The *M×g* field trial was then established in early May 2012, and planted in 3 blocks, with each block divided into 4 m *×* 25 m (5 m *×* 5 m) plots planted at a density of 1 plant m^-2^ (equivalent to 10,000 plants ha^-1^). The plots were randomized within each block to ensure the results were not skewed by environmental conditions (**Supplementary Figure S1**). One of the following four fertilization treatments, consisting of organic and supplementary mineral matter, were added to each plot prior to planting only in year 1:

**D0:** Control plot with no fertilization and no sewage sludge
**D1:** Sewage sludge only
**D2:** Sewage sludge + a single dose of mineral fertilizer (0.5 kg plot^-1^)
**D3:** Sewage sludge + a doubled dose of mineral fertilizer (1 kg plot^-1^)

In October 2011, sewage sludge, defined here as processed human waste, sourced from a municipal sewage treatment plant was applied to each experimental plot at a weight of 1 Mg; this would equate to an addition of organic matter (O.M.) at 400 Mg ha^-1^. Polish legislation states that on agricultural land, sewage sludge is normally applied at a rate of 80–100 Mg ha^-1^. The lack of biologically active layer upon the mine spoil suggested that the normal rates would not be sufficient. The soil was ploghed to a depth of 30 cm and wet sewage sludge was applied by hand as an even spread and mixed with the mine spoil using a tractor and powered cultivator.

The sewage sludge met all guidelines laid out by the Polish legislation in terms of mineral and organic xenobiotics and in terms of sanitary and hygiene standards. The chemical parameters of the sewage sludge were as follows: pH 7.92, dry matter (D.M.) 19.97%, organic carbon (C org.) 336.2 g kg D.M.^-1^, total nitrogen 43.36 g kg D.M.^-1^, C: N ratio of 8:1 and organic matter (O.M.) 600 g kg D.M.^-1^ consisting of 56% C org. Following the mixing of the sewage sludge into the soil, the chemical composition of the soil was as follows: pH 7.42, Corg 4.75 mg kg^-1^, Norg 0.91 mg kg^-1^, and a C:N ratio of 5:1. The additional mineral fertilization was used to test whether the addition of sewage sludge alone is sufficient for the growth and development of *M×g* on such poor soil. For the mineral fertilization, a mix of the Azofoska fertilizer (Azofoska Granules, GRUPA INCO S.A., Poland) was used with the following chemical composition: 13% nitrogen, 19% phosphorus, 16% potassium, 0.18% copper, 0.045% zinc, 0.27% manganese, and 0.09% boron. Fertilization was applied under deep ploghing preceding the field trial.

Over the course of the 3-year period of the study, parameters were evaluated annually. From the center of each plot, six randomly selected plants were collected for analyses: biomass yield (dry matter), plant height and tillering (stem density) were assessed for each plant. The replication of each fertilization mixture treatment gave total of 18 plants for each fertilization treatment. Plant height was measured to the top ligule (excluding the flag leaf) on the tallest stem for each plant; this is very similar to canopy height in *M × giganteus*. For tillering measurements, only stems above 10cm were counted and stems were differentiated from a newly emerged bud by the presence of a ligule leaf. Biomass was harvested during February to March from three successive vegetated periods: 2012–2013, 2013–2014, and 2014–2015.

### Soil Analyses – Analytical Methods

Soil samples were collected by 30 cm corers with a 2 cm diameter. For each plot, a total of five samples were collected; these were then mixed to form one sample before analysis was conducted. Dry mass was assessed in the organic materials (sewage sludge, compost) and in the plant material after drying in a dryer with hot air flow (at 70°C) to constant weight. Total nitrogen was determined after sample mineralization in concentrated sulphuric acid in an open system by Kjeldahl’s (e.g., Bremner, 1960) method using automatic Kjeltec II Plus set (Tecator). Organic carbon content was determined after the sample mineralization in potassium dichromate by Tiurin’s method (Mebius, 1960). Ash component contents in the organic materials and plant samples were assessed after the sample mineralization in a muffle furnace (at 450°C for 5 h) and the ash dissolution in nitric acid. Phosphorus content was determined by vanadium and molybdenum method in Backman DU 640 spectrometer at wavelength 436 nm. Potassium, sodium and calcium were assessed by flame photometry (FES) and magnesium, chromium, and the other heavy metals (only in the organic materials) were determined by atomic absorption spectrometry (AAS) in PU 9100X Phillips apparatus (Ostrowska et al., 1991).

### Statistical Analyses

The data were analyzed by various uni- and multivariate statistical methods (Caliński and Kaczmarek, 1973; Morrison, 1976) in two stages. In stage one a two-factor (three years and four treatments) analysis of variance (ANOVA) was used to test the null hypotheses of no differences between years or between treatments, and, the null hypothesis of no year and treatment interaction.

In stage two, a multivariate analysis of variance was used and a canonical analysis was performed to provide a graphical presentation of treatments with regards to three morphological traits (plant height, tillering and plant biomass yield). The configuration of treatments in the space of the first two canonical variables with the shortest dendrite connecting the points representing those treatments was made. The differences in plant morphology with regards to the differing treatment methods between the first and second years, and, the second and third years were also tested using Duncan’s multiple range test (DMRT) (Gomez and Gomez, 1984). However, configurations of treatments in the space of the first two canonical variables (V_1_ and V_2_) were also performed with regards to increments in the analyzed characteristics of yield for the first year (Y1) and between successive years (Y2–Y1 and Y3–Y2) of the cultivation.

## Results

The *M×g* plants survived the first winter of 2012/2013 with 99% over wintering survival rate. Winter temperatures were not low enough to seriously impact the survival rate; the November to March average temperature at the site was –4°C. Sufficient snowfall provided an insulating layer that helped to protect the vulnerable, first year plants from frost damage. Precipitation total in the growing season (from the beginning of April to the end of October) in the successive years (2012, 2013, and 2014) for the town of Turek was 405, 435, and 460 mm, respectively. Mean temperatures for each growing seasons were 15.4°C, 15.0°C, and 14.9°C.

Results showed that a significant (*P*<0.05) variation in plant traits occurred with regard to the fertilization method (D) as well as the year of analyses (Y). In turn, the interaction between years of analyses and doses of fertilization (Y × D) proved significant only for plant tillering (**Table 1**).

**Table 1:** Results of the two-way analysis of variance for structural traits of *Miscanthus*; plant height, tillering, and plant dry matter yield (^∗^significant at *P* ≤ 0.05).

Source of variation	Df (degrees of freedom)	Mean square		
		Plant height	Tillering	Plant biomass yield
Doses (D)	3	8952.64^∗^	311.92^∗^	10.86^∗^
Year (Y)	2	13322.17^∗^	6249.35^∗^	678.17^∗^
Interaction D × Y	6	317.68	98.44^∗^	3.28
Error	60	324.04	35.58	4.16

**FIGURE 1:**
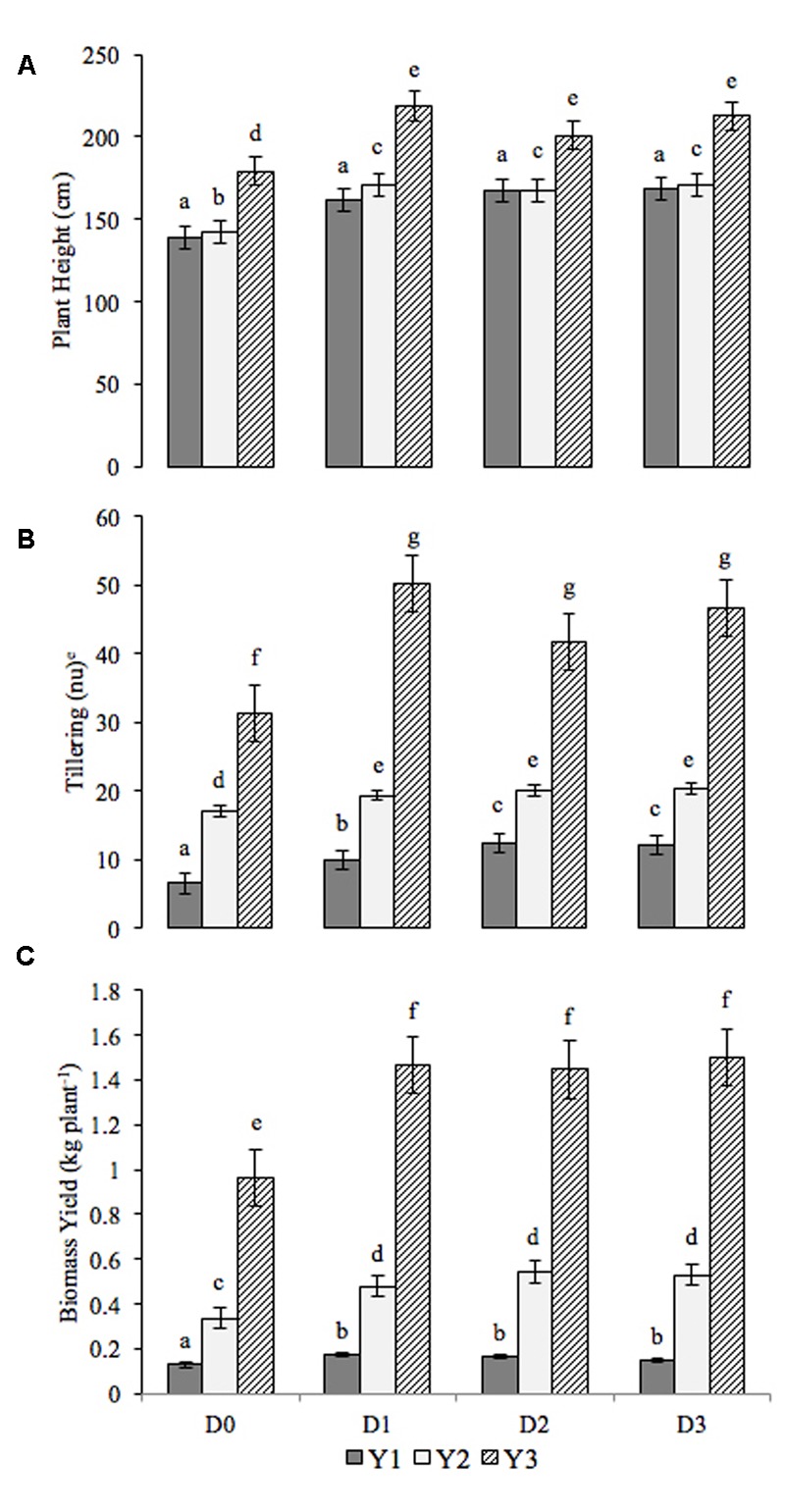
**Plant trait variations over the 3-study period and for the differing fertilization treatments (D0: control, D1: Sewage sludge only, D2: Sewage sludge + single dose of mineral fertilizer, D3: Sewage sludge + double dose of mineral fertilizer).** Y1: Year 1, Y2: Year 2, Y3: Year 3. **(A)** Plant Height, **(B)** Tillering, **(C)** Biomass Yield. The letters above the data series indicated significance differences between the results. Error bars represent standard error.

The variation in plant traits and biomass yield for each year, and, for each fertilization method are displayed in **Figure 1**. Over the 3-year study period, the mean values (derived from the three replications) of plant traits (plant height, tillering and biomass yield) were generally significantly greater (*P* < 0.05) for the fertilized plots (D1, D2, and D3) than the control plot (D0). There are no significant differences between the individual fertilization treatments (D1, D2 and D3) across the individual years other than with regard to tillering. In year one, plots treated with municipal sewage sludge and mineral fertilizer (D2 and D3) yielded a greater number of tillers than those treated only with organic fertilization. There was however, no significant difference in tiller number between the doses (D2 and D3) of mineral fertilizer. By year three, mean plant height in the fertilized plots was 210.61 ± 9.14 cm compared to 179.33 cm in the control, tillering was 46.16 ± 4.27 stems plant^-1^ compared to 31.33 and biomass yield was 1.473 ± 0.28 kg plant^-1^ compared to 0.964 kg plant^-1^ in the fertilized and control plots, respectively.

Since the analyses were conducted across the first 3 years of plant growth and development, and covered the third year where plants might be expected to approach their full yield potential (Greef, 1996; El Bassam, 1997; Deuter and Abraham, 2000; Pude and Jeżowski, 2003; Jeżowski, 2008; Jeżowski et al., 2011); increments of increase in the investigated traits were also analyzed with regards to the fertilization levels.

Between the first and second year of cultivation (Y2–Y1), the greatest increase in plants heights were evident in the plots fertilized with only municipal sewage sludge (D1); the same remains true for the second and third years. Between year two and year three, all fertilized plots showed greater increases in plant height than the control plants. There were no significant differences in plant height relating to the method of fertilization (D1, D2 and D3).

Between year one and year two, the tillering results show that the plants in the control plot grew significantly more stems than those in fertilized plots. The opposite was seen between years two and three, whereby plants treated with fertilizer added significantly more stems than those which had not been treated (mean of 26.33 ± 4.58 stems plant^-1^ compared to the control 14.33 stems plant^-1^). As is the case with plant height, there was no significant difference in tillering between fertilization techniques (D1, D2, and D3). Biomass yield increases were significantly lower between all years in the control plots compared to the fertilized plots. Between years one and two biomass yields were, on average 3.54 ± 0.44 kg plant^-1^ in the fertilized plots compared to 2.08 kg plant^-1^ in the control plots. These figures increased between year two and year three; fertilized plants gained an average of 9.55 ± 044 kg plant^-1^ compared to 6.27 kg plant^-1^ in the unfertilized control plots. Again, no significant differences were evident in the biomass yield between fertilization methods (D1, D2, and D3; **Table 2**).

**Table 2:** Increases in the mean values of plant traits related to different fertilizer doses (D0: control, D1: Sewage sludge only, D2: Sewage sludge + single dose of mineral fertilizer, D3: Sewage sludge + double dose of mineral fertilizer) between the first and second (Y_1_–Y_2_) and second and third (Y_3_–Y_2_) years of the experiment.

Method of fertilization	Year	Plant Trait		
		Plant height (cm)	Tillering (stems number)	Plant biomass yield (kg)
D0	Y_2_–Y_1_	3.33a	10.50b	2.08b
D1		9.35a	9.33a	3.03a
D2		5.00a	7.50a	3.79a
D3		2.62a	8.16a	3.80a
D0	Y_3_–Y_2_	37.17a	14.33b	6.27b
D1		47.98a	30.83a	9.88a
D2		38.34a	21.66a	9.04a
D3		41.49a	26.33a	9.73a

*Superscripts denote significant differences between results (*P* < 0.05).*

**FIGURE 2:**
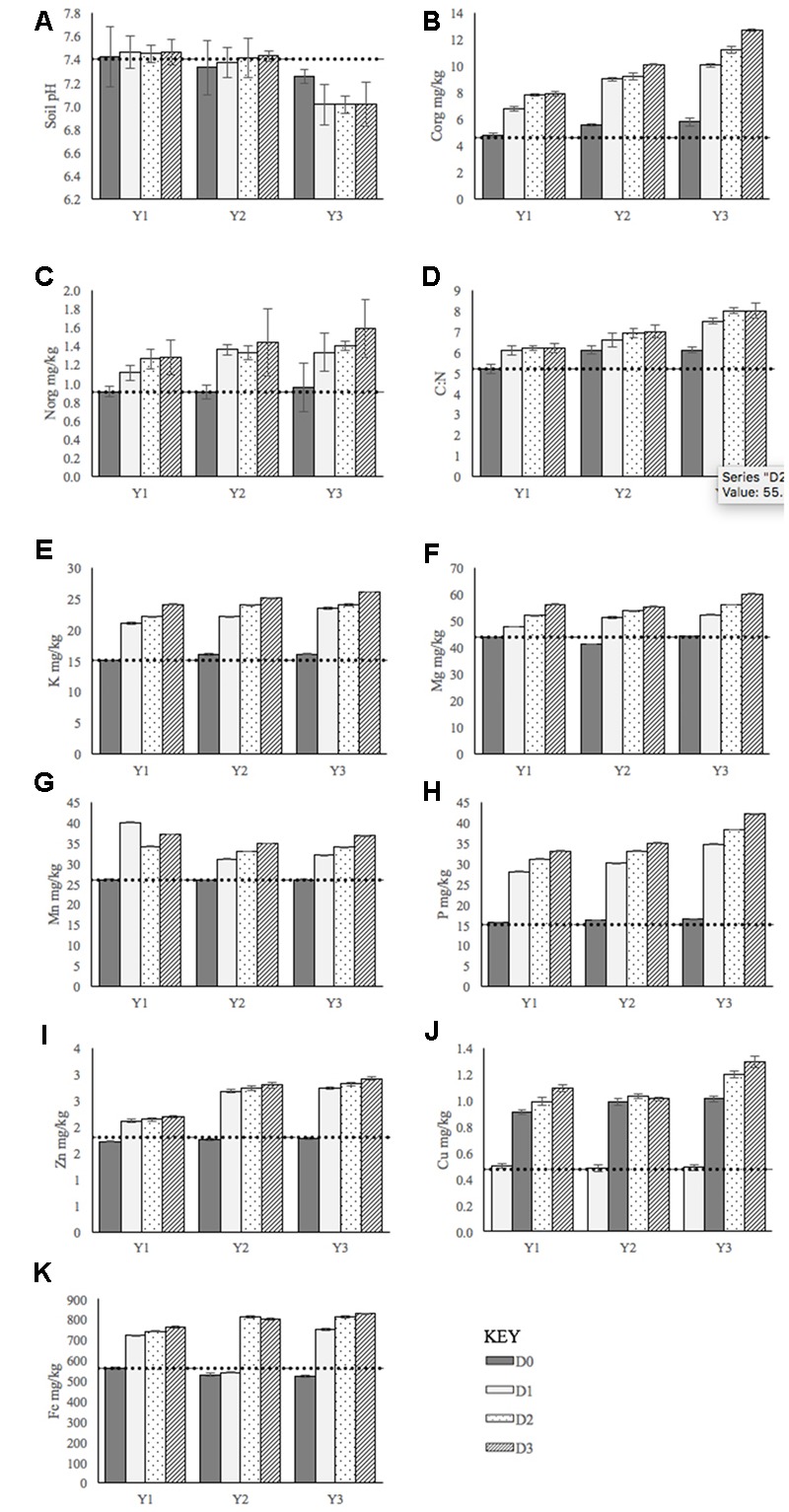
**Soil pH and concentrations of macronutrients throughout the 3-year study period.** Y1: Year 1, Y2: Year 2, Y3: Year 3. **(A)** Soil pH. **(B)** Organic carbon concentration, **(C)** Organic nitrogen concentration, **(D)** Carbon to nitrogen ration, **(E)** Potassium concentration, **(F)** Magnesium concentration, **(G)** Manganese concentration, **(H)** Phosphorus concentration, **(I)** Zinc concentration, **(J)** Copper concentration, **(K)** Iron concentration. In all cases, the dashed line represents soil conditions prior to the study. Error bars represent standard error. Doses of fertilization defined as: D0: control, D1: Sewage sludge only, D2: Sewage sludge + single dose of mineral fertilizer, D3: Sewage sludge + double dose of mineral fertilizer.

A more in-depth interpretation of the recorded results was provided by the application of the analysis of canonical variables V1 and V2. This facilitated a graphic presentation of the results with regards to the effect of individual fertilization methods (D0, D1, D2, and D3) on plant physiology (see Supplementary Data Sheet). This analysis also made it possible to plot a dendrite for the shortest linkages between these doses for the 3-year study period; graphical representations of these results can be found in the Supplementary Information presented with this study. Results of this analysis largely agreed with the ANOVA results previously presented (significant differences in the mean values of morphological and yield traits existed only between control [no fertilizer] and fertilized treatments, and, that there were generally no significant differences between fertilization methods). The only exception to this was observed for the increment of increase in plant tillering between years one and two. The analysis suggested that the application of fertilization treatment D1 (organic fertilization) gave the greatest increase in number of stems in the earlier years when compared to D2 and D3 (organic fertilization supplemented with mineral fertilization). This may also indicate that when *M×g* was approaching its full yielding potential on degraded soils (between years two and three), organic fertilization applied at an adequately high dose is the most effective treatment with regards to tillering. Supplementary mineral fertilization in this later period may have had a lesser effect due to the abundance of essential nutrients (N, P, K, C, and Mg etc.) contained within the organic fertilizer. This implies that additional mineral fertilization had only a slight effect on variation in growth, development and yield (**Figure 2**).

Throughout the study period, soil analysis was conducted to monitor the pH, organic carbon (C org), organic nitrogen (N org) and nutrient content. The pH remained relatively stable in the first 2 years, before decreasing in year three. Perhaps one of the most notable changes was the increasing C org content within the soil across all plots and fertilization doses. The results show that on the control plots (D0), where no fertilization was added, concentrations of C org increased significantly between year one and year three. This indicates that the cultivation of *M×g* naturally supplies carbon to land where organic matter may otherwise be lacking. The concentration of macronutrients within the soil all increased significantly in the fertilized plots, but remained to most extent, at constant pre-experiment levels in unfertilized plots. The most evident exception to this was the levels of Fe; these significantly decreased between years one and three (**Figure 2**).

## Discussion

This current study presents the results of a simple yield and trait development trial investigating the impact of fertilizer treatment on the performance of *M×g* cultivated on reclaimed brown coal mining sites. Whilst many studies report the effect of fertilization of *M×g* on growth, development and yield when cultivated on soils classified as suitable quality for agricultural use (e.g., Greef, 1996; El Bassam, 1997; Munzer, 2000; Pude, 2000), few studies consider the growth on extremely marginal soils. Several studies (e.g., Pogrzeba et al., 2013; Nsanganwimana et al., 2016) assess the cultivation and growth of various species of miscanthus on heavy metal contaminated land, however, little attention is given with regards to degraded (biologically inactive) mine soils, or, plant growth and trait development. In Europe alone, it is estimated that mine spoil and degraded soils cover thousands of hectares (Brown et al., 2003; Augustsson et al., 2015). For this reason, the study presented here may be considered, to a certain degree, pioneering research as it opens new opportunities for the cultivation of energy grasses on extremely marginal soils, otherwise unsuitable for agricultural use (Communication from the Commission to the European Parliament, 2014). If carried out on a large scale, the growth of *M×g* on poor quality land could, in part, satisfy the demands laid out by the European Commission communication (OJ C 163 of 28 May 2014). Growing on such land presents no implications to other arable agriculture and could increase the proportion of perennial crops being utilized within the energy sector. Beyond the production of biomass, the long-term cultivation of *M×g* may also have an advantageous effect on remediation of the degraded soils on which they are grown, restoring their physico–chemical and biological equilibrium. These plants, during both the growing season and harvest, naturally condition the upper layers of degraded soil by supplying organic carbon through leaf drop, biomass residues and root exudates. Additionally, they aid the cycling of many other essential nutrients, e.g., N, P, K, Ca, Na, Mg, Fe, and Si (e.g., El Bassam, 1997; Himken et al., 1997; Majtkowski, 1998; Ercoli et al., 1999; Kahle et al., 2001; Kozak et al., 2006; Danalatos et al., 2007; Kalembasa and Malinowska, 2007; Borzącka-Walker, 2008; Christian et al., 2008; Curley et al., 2009). The ability of *M×g* to supply carbon to the soil is demonstrated within this study; organic carbon levels significantly increased in the soil following the cultivation of the plants even without fertilization. The concentrations of most macronutrients remained relatively stable in plots where no fertilizer was added, suggesting that whilst the crop may aid in the cycling of nutrients, in most cases, it does not significantly affect the concentrations.

The results indicate that crop performance on brown coal mining sites was significantly enhanced by the application of fertilizer. The best growth, development and yields of *M×g* over a period of 3 years (from planting to reaching full yield by plants in the third year) were achieved by a very high dose of organic fertilization (approximately 400 Mg fresh matter ha^-1^) contained, for example, in municipal sewage sludge. Supplemental mineral fertilization in this, case showed no significant effect. These analyses also showed that by the third year of establishment, the plants yielded around 1.5 kg D.M. plant^-1^ (∼15 Mg D.M. ha^-1^ at a density of 10,000 plants ha^-1^). The results suggest that the nutrient content in the sewage sludge alone was sufficient to fulfill the requirements of *M×g*. *M×g* is highly efficient with regards to nitrogen use, and as such, is typically unresponsive (in terms of harvestable yield at least) to concentrations of mineralized N above 50 kg ha^-1^ (McCalmont et al., 2017). There were no significant differences in yield ha^-1^ between the mineral fertilization doses (D1, D2, and D3). This would suggest that demand for N had been satisfied by existing mineral levels in the soil along with the nitrification of organic N added in the sewage sludge.

Contempory studies (Hastings et al., unpublished) discuss the economic viability of growing *M×g.* Current harvest prices in the United Kingdom equate to ∼ ¬89 [exchange rate correct at time of writing] Mg^-1^ for bales with <14% moisture content. Based on this price, and the yields achieved within this study, biomass from 1 ha of *M×g* grown on post mining land would have a value of ¬1330. The cost typical of rhizome propagation in the United Kingdom is ¬2364–2956 ha^-1^ (Terravesta, personal communication), however, on marginal post mining soils this cost will be higher due to the preliminary work needed to establish a suitable organic soil. Harvesting costs also need to be considered; Hastings et al. (unpublished) suggest these to be ¬48.09 Mg^-1^ for a ∼13 ha field used within their study. Commercial prices are considerably cheaper; ∼ ¬29.70 Mg^-1^ (Terravesta, personal communication). Using the yields (15 Mg ha^-1^) of this study, this would equate to harvesting costs of ¬436 - ¬721 ha^-1^ for the commercial and experimental estimates, respectively. Assuming that harvest starts in year 3, and using the more expensive experimental costing, after year 3 the overall cost incurred (propagation cost [¬2956] + harvest cost [¬721] – biomass value [¬1330]) would be ∼ ¬2347 ha^-1^. After initial planting there are no further establishment costs and the crop requires few inputs. Thus, if the biomass value and harvest costs remain constant, this figure decreases by ¬609 (biomass value – harvesting costs) each year. Therefore, using the figures provided, a plantation on reclaimed soil could be profitable after the seventh year of establishment. However, due to the nature of the land (unstable and undulating terrain), conventional forage harvest methods may have to be adapted to cut the crop at a greater height (15–20 cm rather than 10 cm). This would result in a biomass yield decrease of ∼0.5 Mg ha^-1^. As such, it is more plausible to suggest that such a plantation would become profitable after year 10. It should be stressed here, that whilst the establishment of *M×g* on post mining land could be profitable, its role in the reclamation of land is of great significance. This study shows that following establishment, organic content in the soil increases. Therefore, a long-term plantation (15 to 20 years) could significantly contribute to the reclamation of extremely degraded soils, on which other economically important plant species maybe grown in the future (Ussiri and Lal, 2014; Lord, 2015). Although the process of reclamation by the growth of *M×g* may take longer than other conventional methods, the costs associated with the process maybe greatly reduced. Estimates suggest remediation can range from ¬147,774–¬472,876 ha^-1^ for post mining waste (English Partnerships, 2008).

The results of this study indicate that *M×g* shows great growth potential on land that is unsuitable for other agricultural uses. However, there are a number of factors to be considered. The dose of organic matter in the form of sewage sludge was four times that of normal agricultural application, due to the poor nature of the soil. Given the yields of the control plots (∼0.9 kg plant^-1^ which equates to ∼9 Mg ha^-1^), it is possible that viable yields could be achieved with much lower doses of organic fertilizer. Increasing plant density may also increase the yields of biomass per hectare. These factors are potential avenues for future work. Furthermore, the quality of the biomass produced by plants cultivated on degraded land would need to be assessed prior to commercial and economic use. If the harvested biomass was to contain elevated levels of heavy metals, it would be unsuitable for combustion as this would result in the slagging and corrosion of biomass boilers (Obernberger, 1998). However, should this be the case, there are other possible feedstock applications, such as anaerobic digestion (a process where the digestate encompassing the contaminants can be contained), that could be considered (e.g., Kiesel and Lewandowski, 2015).

## Author Contributions

MM and SJ contributed equally to this work as the first authors (60% contributions in experimental design, data collections, and manuscript writing). MM work leader. JC-W, SB, and SO – 5% each contribution in data analyses and manuscript writing. ZK, WO, and AM – 5% each contribution in data analyses, experimental design, data collections, and manuscript writing. JM – 10% contribution in data analyses and manuscript writing.

## Conflict of Interest Statement

MM holds executive position at the time of running this experiment and has financial interest in Energene Sp. z.o.o. SB holds a technical position in Terravesta Ltd. The other authors declare that the research was conducted in the absence of any commercial or financial relationships that could be construed as a potential conflict of interest.

## Supplementary Material

FIGURE S1 **The randomised experimental field trial design**.

## References

[B1] Augustsson A. L. M. Uddh-Söderberga T. E. Hogmalmb K. J. Filipssona M. E. M. (2015). Metal uptake by homegrown vegetables – The relative importance in human health risk assessments at contaminated sites. *Environ. Res* 138 181–190. 10.1016/j.envres.2015.01.020 25723126

[B2] Borzącka-Walker M. (2008). *Produkcyjnośæ Miskanta (Miscanthus ssp.) w Różnych Warunkach Siedliskowych i Pogodowych [Productivity of Miscanthus ssp. under Different Habitat and Weather Conditions].* Doctoral dissertation, Institute of Soil Science and Plant Cultivation SRI, Puławy 1–105.

[B3] Bremner J. M. (1960). Determination of nitrogen in soil by the Kjeldahl method. *J. Agric. Sci.* 55 11–33. 10.1017/S0021859600021572

[B4] Brown S. L. Henry C. L. Chaney R. L. Compton H. DeVolder P. (2003). Using municipal biosolids in combination with other residuals to restore metal-contaminated mining areas. *Plant Soil* 249 203–215. 10.1023/A:1022558013310

[B5] Caliński T. Kaczmarek Z. (1973). “Metody kompleksowej analizy doświadczenia wielocechowego [Methods of comprehensive analysis of multivariate experiment],”in *Proceedings of the Third Colloquium on Methodology in Agrobiometry, PAN i PTB* Warszawa 258–320.

[B6] Christian D. C. Riohe A. B. Yates N. E. (2008). Growth, yield and mineral content of *Miscanthus × giganteus* grown as a biofuel for 14 successive harvest. *Ind. Crops Prod.* 28 320–327. 10.1016/j.indcrop.2008.02.009

[B7] Chung J. H. Kim D. S. (2012). *Miscanthus* as a potential bioenergy crop in East Asia. *J. Crop Sci. Biotechnol.* 15 65–77. 10.1007/s12892-012-0023-0

[B8] Clifton-Brown J. Robson P. Davey C. Farrar K. Hayes C. Huang L. (2013). “Breeding Miscanthus for bioenergy,” in *Bioenergy Feedstocks: Breeding and Genetics* eds Saha M. Bhandari H. S. Bouton J. H. (Hoboken, NJ: John Wiley & Sons, Inc) 67–81. 10.1002/9781118609477.ch5

[B9] Clifton-Brown J. C. Lewandowski I. Andersson B. Basch G. Christian D. G. Kjeldsen J. B. (2001). Performance of 15 *Miscanthus* genotypes at five sites in Europe. *Agron. J.* 93 1013–1019. 10.2134/agronj2001.9351013x

[B10] Communication from the Commission to the European Parliament (2014). A policy framework for climate and energy for the period from 2020 to 2030. Brussels: European Commission 1–27.

[B11] Curley E. M. O’ Flynn M. G. McDonell K. P. (2009). Nitrate leaching losses from *Miscanthus × giganteus* impact on groundwater quality. *J. Agron.* 8 107–112. 10.3923/ja.2009.107.112

[B12] Danalatos N. G. Archontoulis S. V. Mitsios I. (2007). Potential growth and biomass productivity of *Miscanthus × giganteus* as affected by plant density and N-fertilization in central Greece. *Biomass Bioenergy* 31 145–152. 10.1016/j.biombioe.2006.07.004

[B13] Deuter M. Abraham J. (2000). *Wiessenstand in der Miscanthus*. Universtat in Bonn. Beitrage zu agrarwissenschaften. *Band* 19 8–14.

[B14] Deuter M. Jeżowski S. (1998). Szanse i problem hodowli traw z rodzaju *Miscanthus* jako roślin alternatywnych [Chances and problems for growing grasses from the genus Miscanthus as alternative plants]. *Hodowla Nasiennictwo* 4 45–48.

[B15] Deuter M. Jeżowski S. (2000). Breeding conditions of the giant grasses Miscanthus genus Post. *Nauk Rol.* 2 59–67.

[B16] El Bassam N. (1997). Renewable energy. *REU Tech. Ser.* 46 4–196.

[B17] English Partnerships (2008). *Contamination and Dereliction Remediation Costs.* Available at: http://www.regenerate.co.uk/EP_Contamination%20&%20Remediation%20costs.pdf [accessed February 23, 2017].

[B18] Ercoli L. Mariotti A. Masoni A. Bonari E. (1999). Effect of Irrigation and Nitrogen Fertilization on Biomass Field and Efficiency of Energy Use in Crop Production of *Miscanthus*. *Field Crops Res.* 63 3–11. 10.1016/S0378-4290(99)00022-2

[B19] Faber A. Borek R. Borzęcka–Walker M. (2007). Szacunek sekwestracji wêgla w uprawach roślin energetycznych miskanta i wierzby [Estimated carbon sequestration in cultures of energy crops Miscanthus and willow]. *Acta Agrophys.* 4 84–89.

[B20] Faber A. Kuś J. (2009). *Produkcja roślinna na cele energetyczne a racjonalne wykorzystanie rolniczej przestrzeni produkcyjnej Polski [Plant production for energy purposes and rational use of agricultural production space in Poland].* Puławy: Wyd. IUNG-PIB 63–75.

[B21] FAO (1999). *CGIAR Research Priorities for Marginal Lands. Consultative Group on International Agricultural Research (CGIAR).* Available at: http://www.fao.org/Wairdocs/TAC/X5784E/x5784e00.htm#Contents [accessed January 17, 2013].

[B22] Gomez K. A. Gomez A. A. (1984). *Statistical Procedures for Agricultural Research.* New York, NY: Wiley 1–100.

[B23] Greef J. M. (1996). Etablirung und Biomassebildung von *Miscanthus × giganteus*. Gotingen: Cuvillier Verlag 1–162.

[B24] Greef J. M. Deuter M. (1993). Syntaxonomy of *Miscanthus × giganteus* GREEF et DEU. *Angewandte Botanik* 67 87–90.

[B25] Hansen E. M. Christensen B. T. Jensen L. S. Kristensen K. (2004). Carbon sequestration in soil beneath long-term Miscanthus plantations as determined by 13C abundance. *Biomass Bioenergy* 26 97–105. 10.1016/S0961-9534(03)00102-8

[B26] Hastings A. Clifton-Brown J. Wattenbach M. Mitchell P. Stampfl P. Smith P. (2009). Future energy potential of *Miscanthus* in Europe. *Glob. Change Biol. Bioenergy* 1 180–196. 10.1111/j.1757-1707.2009.01012.x

[B27] Hastings A. Clifton-Brown J. Wattenbach M. Stampfel P. Mitchell P. Smith P. (2008). Potential of Miscanthus grasses to provide energy and hence reduce greenhouse gas emissions. *Agron. Sustain. Dev.* 28 465–472. 10.1051/agro:2008030

[B28] Heaton E. A. Clifton-Brown J. Voigt T. B. Jones M. B. Long S. P. (2004). Miscanthus for renewable energy generation: European Union experience and projections for Illinois. *Mitig. Adapt. Strategies Glob. Change* 9 433–451. 10.1023/B:MITI.0000038848.94134.be

[B29] Himken M. Lammel J. Neukirchen D. Czypionka–Krause U. Olfs H. (1997). Cultivation of *Miscanthus* under West European conditions: seasonal changes in dry matter production, nutrient uptake and remobilization. *Plant Soil* 189 117–126. 10.1023/A:1004244614537

[B30] Jeżowski S. (1994). *Miscanthus sinensis* ‘Giganteus’- a grass for industrial and energetic purpose. *Genet. Pol.* 35A 372–337.

[B31] Jeżowski S. (2001). Rośliny energetyczne - ogólna charakterystyka, uwarunkowania fizjologiczne i znaczenie w produkcji ekopaliwa [Energy crops – general characteristics, physiological condiitons and role in ecofuel production]. *Post. Nauk. Roln.* 2 19–27.

[B32] Jeżowski S. (2008). Yield traits of six clones of Miscanthus in the 3 years following planting in Poland. *Ind. Crop. Prod.* 27 65–68. 10.1016/j.indcrop.2007.07.013

[B33] Jeżowski S. Głowacka K. Kaczmarek Z. (2011). Variation in biomass yield and morphological traits of energy grasses from the genus *Miscanthus* during the first years of crop establishment. *Biomass Bioenergy* 35 814–821. 10.1016/j.biombioe.2010.11.013

[B34] Kahle P. Beuch S. Boelcke B. Leinweber P. Schulten H. (2001). Cropping Miscanthus in central Europe: biomass production and influence on nutrients and soil organic matter. *Eur. J. Agron.* 15 171–184. 10.1016/S1161-0301(01)00102-2

[B35] Kalembasa D. Malinowska E. (2007). Wpływ dawek osadu ściekowego na plon i skład chemiczny trawy *Miscanthus sacchariflorus* [The effect of sewage sludge doses on yield and chemical composition of grass *Miscanthus sacchariflorus*]. *Fragm. Agron.* 1 113–117.

[B36] Kiesel A. Lewandowski I. (2015). Miscanthus as biogas substrate-Cutting tolerance and potential for anaerobic digestion. *GCB Bioenergy* 9 153–167. 10.1111/gcbb.12330

[B37] Kozak K. Kotecki A. Dobrzański Z. (2006). The effect of nitrogen fertilisation on growth and yield of *Miscanthus giganteus*. *Chem. Agric.* 7 209–227.

[B38] Lewandowski I. (2006). “Miscanthus - a multifunctional biomass crop for the future,” in *Alternative Plants for Sustainable Agriculture* eds Jeżowski S. Wojciechowicz K. M. Zenkteler E. (Poznań: Institute of Plant Genetics PAS) 83–90.

[B39] Lewandowski I. Clifton-Brown J. C. Scurlock J. M. O. Huisman W. (2000). Miscanthus: European experience with a novel energy crop. *Biomass Bioenergy* 19 209–227. 10.1016/S0961-9534(00)00032-5

[B40] Lord R. A. (2015). Reed canarygrass (*Phalaris arundinacea*) outperforms Miscanthus or willow on marginal soils, brownfield and non-agricultural sites for local, sustainable energy crop production. *Biomass Bioenergy* 78 110–125. 10.1016/j.biombioe.2015.04.015

[B41] Majtkowski W. (1998). Przydatnośæ wybranych gatunków traw typu C4 do upraw alternatywnych w Polsce [Suitability of selected C4 grasses for alternative cultures in Poland]. *Hod. Ros. Nas.* 2 41–48.

[B42] McCalmont J. P. Hastings A. McNamara N. P. Richter G. M. Robson P. Donnison I. S. (2017). Environmental costs and benefits of growing Miscanthus for bioenergy in the UK. *GCB Bioenergy.* 9 489–507. 10.1111/gcbb.12294 28331551 PMC5340280

[B43] Mebius L. (1960). A rapid method for the determination of organic carbon in soil. *Anal. Chim. Acta* 22 120–124. 10.1016/S0003-2670(00)88254-9

[B44] Morrison D. F. (1976). *Multivariate Statistical Methods* 2nd Edn. Tokyo: McGraw-Hill 1–56.

[B45] Munzer W. (2000). Rhizompflanzen, Alternative? Beitrage zu Agrarwissenschaften. *Unvarstat Bonn* 19 15–20.

[B46] Nsanganwimana F. Waterlot C. Louvel B. Pourrut B. Douay F. (2016). Metal, nutrient and biomass accumulation during the growing cycle of Miscanthus established on metal-contaminated soils. *J. Plant Nutr. Soil Sci.* 179 257–269. 10.1002/jpln.201500163

[B47] Obernberger I. (1998). Decentralized biomass combustion: state of the art and future development. *Biomass Bioenergy* 14 33–56. 10.1016/S0961-9534(97)00034-2

[B48] Ostrowska A. Gawliński S. Szczubialka Z. (1991). *Metody analizy i oceny właściwości gleb i roślin-katalog.* Warszawa: Wydawnictwo IOŚ.

[B49] Pogrzeba M. Krzyżak J. Sas-Nowosielska A. (2013). *Environmental Hazards Related to Miscanthus × giganteus Cultivation on Heavy Metal Contaminated Soil*. *E3S Web Conf.* 1:29006 10.1051/e3sconf/20130120006

[B50] Pude R. (2000). “Anbau und rttrage von Miscanthus in Europa. Materiały konferencyjne,” in Proceedings of the Polsko-niemiecka Konferencja na Temat Wykorzystania Trzciny Chiñskiej Połczyn-Zdrój 91–95.

[B51] Pude R. Jeżowski S. (2003). Effect of selected morphogenetic traits on growth and development of *Miscanthus* ssp. *Biuletyn IHAR* 227 573–583.

[B52] Sacks E. J. Juvik J. A. Lin Q. Stewart R. Yamada T. (2013). “The gene pool of *Miscanthus* species and its improvement,” in *Genomics of the Saccharinae* ed. Paterson A. H. (New York, NY: Springer) 73–101.

[B53] The European Parliament and the council of the European Union (2009). Directive 2009/28/EC of the European Parliament and of the council. *Offic. J. Euro. Union* L140 16–62.

[B54] Ussiri D. A. Lal R. (2014). Miscanthus agronomy and bioenergy feedstock potential on minesoils. *Biofuels* 5 741–770. 10.1080/17597269.2015.1024388

[B55] Wanat N. Austruy A. Joussein E. (2013). Potentials of *Miscanthus × giganteus* grown on highly contaminated Technosols. *J. Geochem. Explor.* 126–127 78–84. 10.1016/j.gexplo.2013.01.001

