# Supplementary figures and images for: Establishment, Growth, and Yield Potential of the Perennial Grass Miscanthus × Giganteus on Degraded Coal Mine Soils
Source: Front Plant Sci. 2017 Jun 12;8:726. doi: 10.3389/fpls.2017.00726 (PMC5466985; doi:10.3389/fpls.2017.00726)

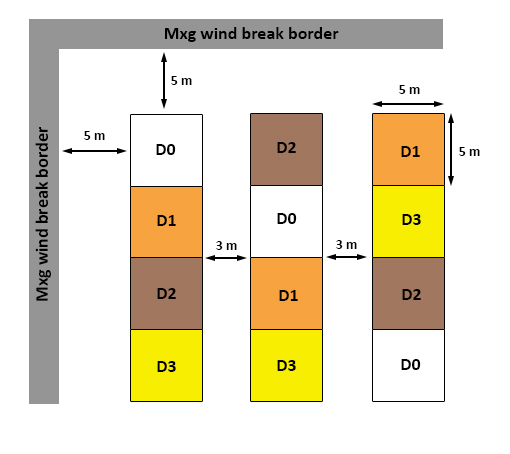

Supplement: FIGURE S1 — The randomised experimental field trial design. [file Image_1.JPEG]
